# A newly developed paper embedded microchip based on LAMP for rapid multiple detections of foodborne pathogens

**DOI:** 10.1186/s12866-021-02223-0

**Published:** 2021-06-28

**Authors:** Mimi Zhang, Jinfeng Liu, Zhiqiang Shen, Yongxin Liu, Yang Song, Yu Liang, Zhende Li, Lingmei Nie, Yanjun Fang, Youquan Zhao

**Affiliations:** 1 College of Precision Instrument and Optoelectronics Engineering, Tianjin University, Tianjin, 300072 China; 2 Tianjin Institute of Health and Environmental Medicine, A Key Laboratory of Risk Assessment and Control for Environment and Food Safety, Tianjin, 300050 China

**Keywords:** Paper-based microchip, Rapid detection, LAMP, Foodborne pathogens, Calcein fluorescence

## Abstract

**Background:**

Microfluidic chip detection technology is considered a potent tool for many bioanalytic applications. Rapid detection of foodborne pathogens in the early stages is imperative to prevent the outbreak of foodborne diseases, known as a severe threat to human health. Conventional bacterial culture methods for detecting foodborne pathogens are time-consuming, laborious, and lacking in pathogen diagnosis. To overcome this problem, we have created an embedded paper-based microchip based on isothermal loop amplification (LAMP), which can rapidly and sensitively detect foodborne pathogens.

**Results:**

We embed paper impregnated with LAMP reagent and specific primers in multiple reaction chambers of the microchip. The solution containing the target pathogen was injected into the center chamber and uniformly distributed into the reaction chamber by centrifugal force. The purified DNA of *Escherichia coli O157:H7, Salmonella spp., Staphylococcus aureus, and Vibrio parahaemolyticus* has been successfully amplified and directly detected on the microchip. The *E. coli O157:H7* DNA was identified as low as 0.0134 ng μL^− 1^. Besides, the potential of this microchip in point-of-care testing was further tested by combining the on-chip sample purification module and using milk spiked with *Salmonella spp*.. The pyrolyzed milk sample was filtered through a polydopamine-coated paper embedded in the inside of the sample chamber. It was transported to the reaction chamber by centrifugal force for LAMP amplification. Then direct chip detection was performed in the reaction chamber embedded with calcein-soaked paper. The detection limit of *Salmonella spp*. in the sample measured by the microchip was approximately 12 CFU mL^− 1^.

**Conclusion:**

The paper embedded LAMP microchip offers inexpensive, user-friendly, and highly selective pathogen detection capabilities. It is expected to have great potential as a quick, efficient, and cost-effective solution for future foodborne pathogen detection.

## Background

As pointed out by the World Health Organization (WHO), foodborne pathogens are the main threat to food safety and lead to foodborne disease outbreaks [1]. There are 200,000,000 foodborne diarrhea cases globally each year, including 65% or more of cases originating from foodborne pathogens [2]. Annual global statistics show that 1.8 million people die of intestinal diseases each year [3]. Therefore, there is an urgent need to screen foodborne pathogens for clinical diagnosis rapidly foodborne pathogens for clinical diagnosis [4]. Conventional microbial detection methods depend upon culturing organisms in selective media followed by microbial identification employed morphological, biochemical, or immunological characteristics [5]. However, these methods typically require 3 to 5 days to confirm pathogenic microorganisms [6]. This time frame does not meet the need for rapid clinical diagnosis or identification in field food security investigations, especially in cases of acute infection. Therefore, it is essential to develop a rapid and sensitive method that can be broadly applied for detecting foodborne pathogenic microorganisms.

Recently, detection methods based on nucleic acid amplification have been introduced to identify susceptible and selective pathogens [7]. These methods mainly focused on polymerase chain reaction (PCR) [8, 9], rolling circle amplification (RCA) [10], helicase-dependent amplification (HDA) [11], nucleic acid sequence-based amplification (NASBA) [12], recombinant enzyme polymerase amplification (RPA) [13, 14], and LAMP [15, 16]. LAMP is an up-and-coming pathogen detection technology that relies on a set of oligonucleotide primers to identify the target of interest by the increment of Bst DNA polymerase displacement activity [17, 18]. One advantage of LAMP is its ability to amplify nucleic acid sequences with high sensitivity and selectivity under isothermal conditions (60–65 °C) [19]. However, conventional LAMP reactions involve multiple wet-bench operations and the use of expensive equipment. Microfluidic technology can overcome this limitation of conventional LAMP schemes, and the most recent LAMP technologies provide significant improvements by reducing reagent consumption and reaction time [20, 21].

LAMP synthesizes a large amount of byproduct pyrophosphate, which can be considered as an indicator of the success of the LAMP reaction. In the LAMP reaction, pyrophosphate ions react with magnesium (Mg^2+^) ions to form a precipitate. For LAMP detection, many indirect techniques have been proposed to monitor pyrophosphate. For example, a turbidimeter is used for turbidity measurement [17], hydroxy naphthol blue (HNB) is used for colorimetric detection [22], and a chelating agent such as calcein is used for fluorescence detection [23]. Here we chose calcein with high sensitivity and low cost for LAMP detection.

Because of the vast advantages of the LAMP reaction, many efforts have been made to integrate it into a microfluidic device for foodborne pathogen detection [24–26]. Jin et al. developed a self-priming compartmentalization polymethyl methacrylate (SPC PMMA) chip to visualize the detection of foodborne pathogens. However, a DNA extraction kit was required to extract the DNA templates [27]. Hongwarittorrn et al. proposed a paper analysis device for semi-quantitative LAMP products, but the LAMP reaction was carried out in a tube [28]. Guo et al. designed a LAMP-based microfluidic chip that integrated this method for bacterial detection. Using this device, the bacterial DNA was isolated by binding to the silica beads, amplified the target genes by LAMP, and directly detected products on the same chip by coloring the calcein. Although the method could perform many steps in one format for identifying bacteria, it required complicated processes for operation [29].

Polydopamine is polymerized from dopamine under alkaline and oxidizing conditions. It is a biocompatible material with a wide range of versatility, can coat various surfaces, and form nanoparticles (NPs) [30]. Polydopamine usually has two typical forms: film and NPs. Due to its extraordinary adhesive force, polydopamine films can be coated over various surfaces by oxidative polymerization of dopamine. If there is no surface to accommodate or intermediate polydopamine aggregation, polydopamine oligomers aggregate together to form polydopamine NPs, usually in the form of nanospheres [31]. DNA aptamers specifically bind dopamine. When dopamine is polymerized to form polydopamine, some structural elements (such as aromatic rings, catechol, and amines) are partly conserved. Although the structure of polydopamine is very different from that of dopamine, the presence of high-affinity aptamers suggests the feasibility of DNA adsorption on polydopamine [32]. The PDA material shows excellent hydrophilicity and biocompatibility because it has many functional groups (catechol hydroxyl, amino, imine, quinone) that can react with many molecules, which will be beneficial for DNA extraction [31].

The original microfluidic chip processing technology originated from micro-electromechanical systems (MEMS) processing technology. The design and processing cost of a microfluidic chip is very high, seriously hindering their analytical chemistry and life sciences application. Today, single microfluidic chips made of standardized glass or polymer materials and produced by microfluidic technology companies in Europe and the United States cost between tens of dollars to hundreds of dollars. We chose polycarbonate (PC) as the chip material and processed the chip by laser ablation to develop a lower-cost microchip device. Simultaneously, the cost of fluorescent detection reagents (calcein) and the UV Analyzer is also meager.

Recently, centrifugal force injection has been widely used [33]. This injection mode can divide the liquid volume into several smaller volumes for different analytical reactions because it only requires a rotating motor to make the sample flow through. The rotation speed can control the injection. We also use the centrifugal force injection mode to quickly and easily control the fluid flow in the micro-device.

This study developed a paper-embedded microchip for multiple detections of foodborne pathogens. In particular, a polydopamine-coated paper was used to purify the genomic DNA (gDNA) of *S. aureus* from the milk sample. Low-cost calcein was used for indirect detection of the LAMP procedure. Rapid and parallel screening of multiple pathogens (*E. coli O157:H7, Salmonella spp., S. aureus,* and *V. parahaemolyticus*) was performed individually. Besides, we analyzed the sensitivity and specificity of microchips and provided an optimized technology to detect foodborne pathogens.

## Results

### Optimizing LAMP reaction conditions

**Fig. 1:**
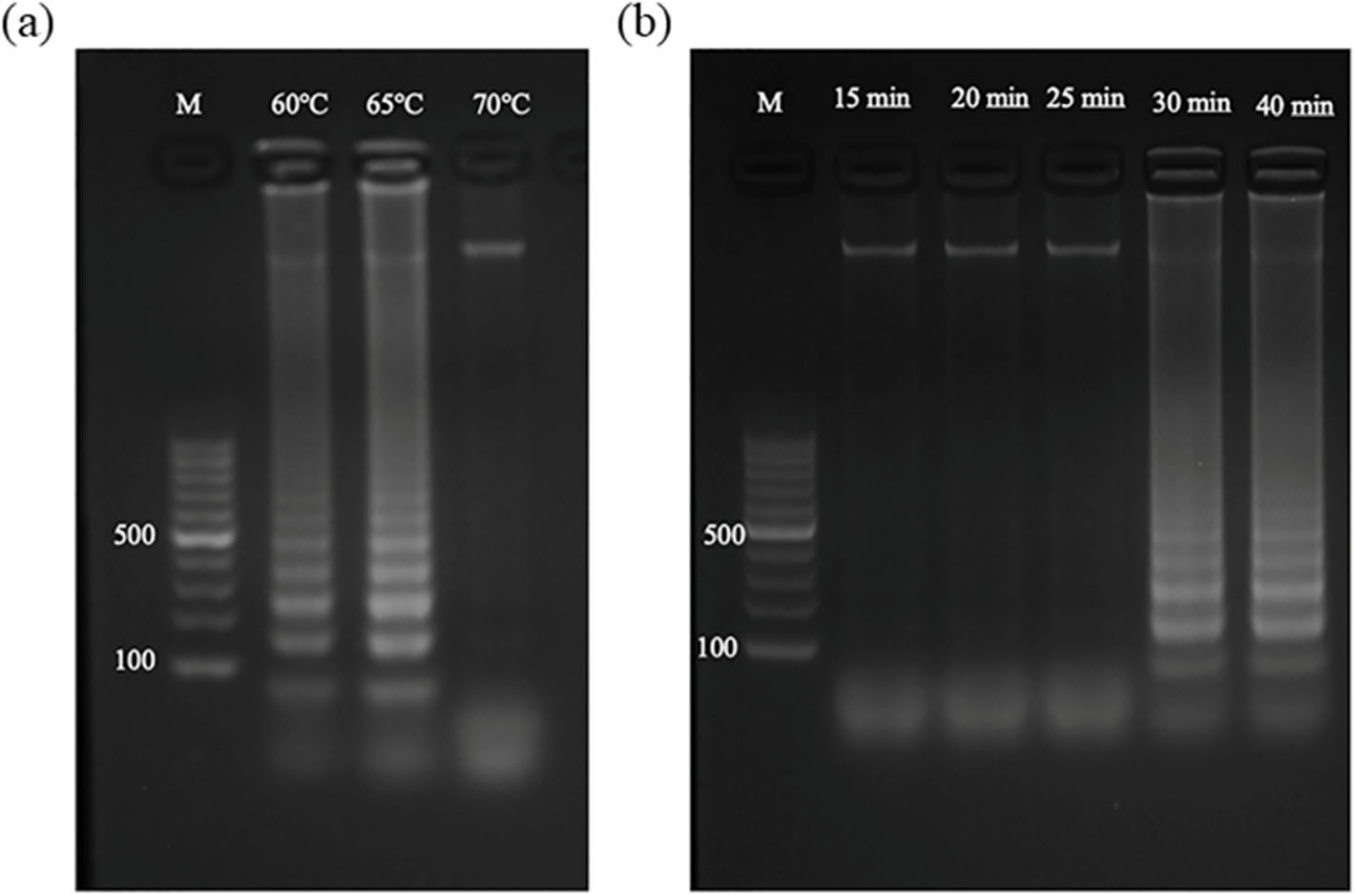
Results of optimizing LAMP reaction conditions. **a** Optimization of reaction temperature: the lane #1 ~ #4 is respectively the electrophoresis result of the market, or at 60 °C, 65 °C, and 70 °C; **b** Optimization of reaction time: the lane #1 ~ #4 is respectively the electrophoresis result of market or at 15 min, 20 min, 25 min, 30 min, and 40 min

To optimize the conditions of the LAMP reaction, firstly, the reaction temperature was set to 60 °C, 65 °C, and 70 °C, and the reaction time was set to 15 min, 20 min, 25 min, 30 min, and 40 min. Figure 1a. Shows electrophoresis results for LAMP at different reaction temperatures. These results clearly show that there is no scalariform band at 70 °C. However, there are scalariform bands at 60 °C and 65 °C, and the latter is more evident and brighter than that of 60 °C. Figure 1b shows the electrophoresis results of LAMP at different reaction times, which indicates that there are no scalariform bands in the first three lanes, but the bands from the fourth lane become clear and bright.

**Fig. 2:**
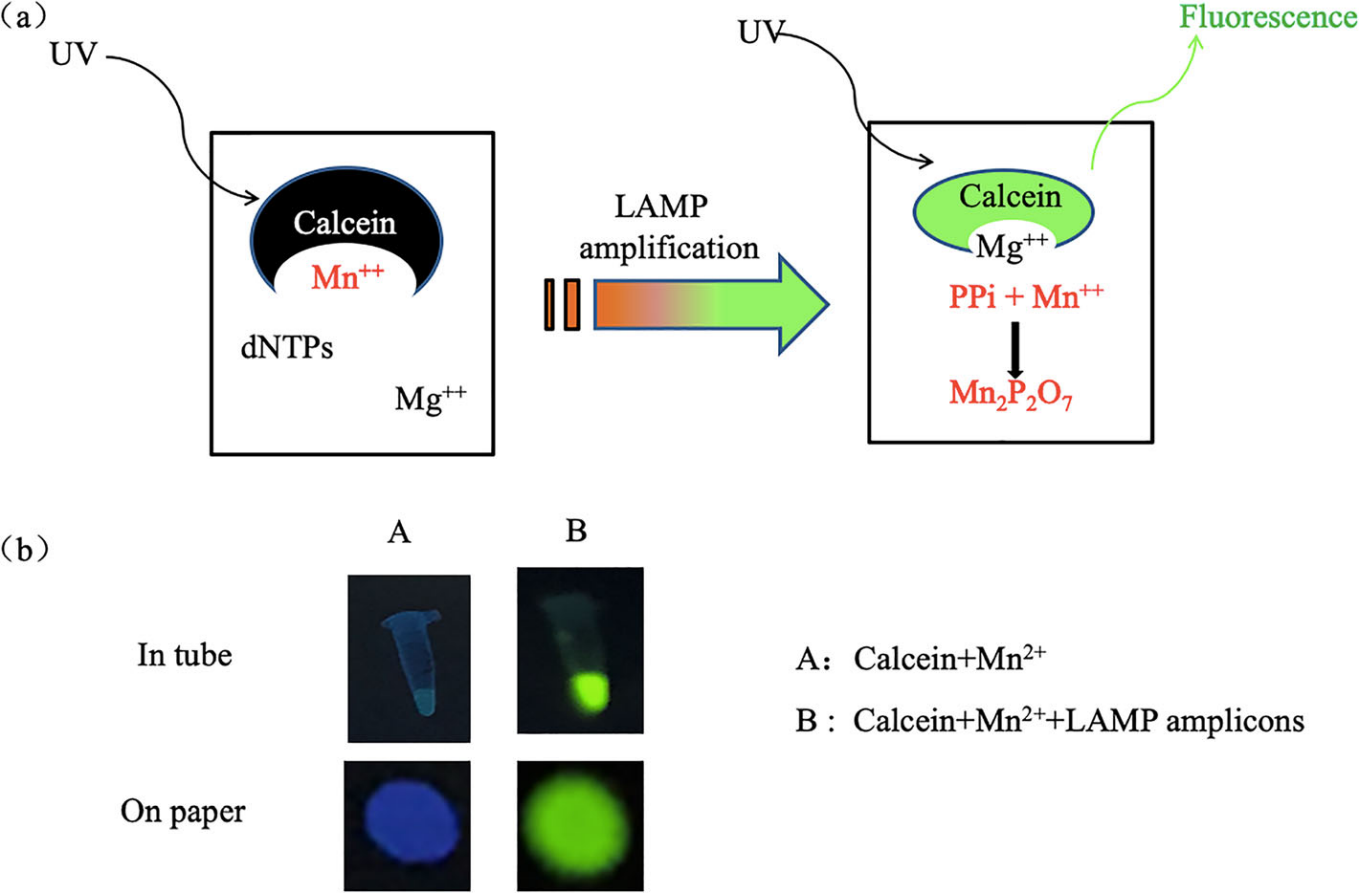
Testing of the fluorescence signal. **a** Inspection mechanism of calcein, **b** Change in solution color when pyrophosphate ion exists after LAMP reaction

As mentioned before, calcein can detect the products of LAMP reaction, for which we chose calcein as the detection reagent in this study. To test its reliability, we had figured the following experiments. Figure 2a shows the reaction principle using calcein to test LAMP amplicons. Figure 2b shows the results of the calcein method for LAMP testing with heating at 65 °C for 30 min. In the reaction mixture, strong fluorescence can be observed in the presence of the DNA template. However, the fluorescence signal is invisible without the DNA template.

### Microchip operation

**Fig. 3:**
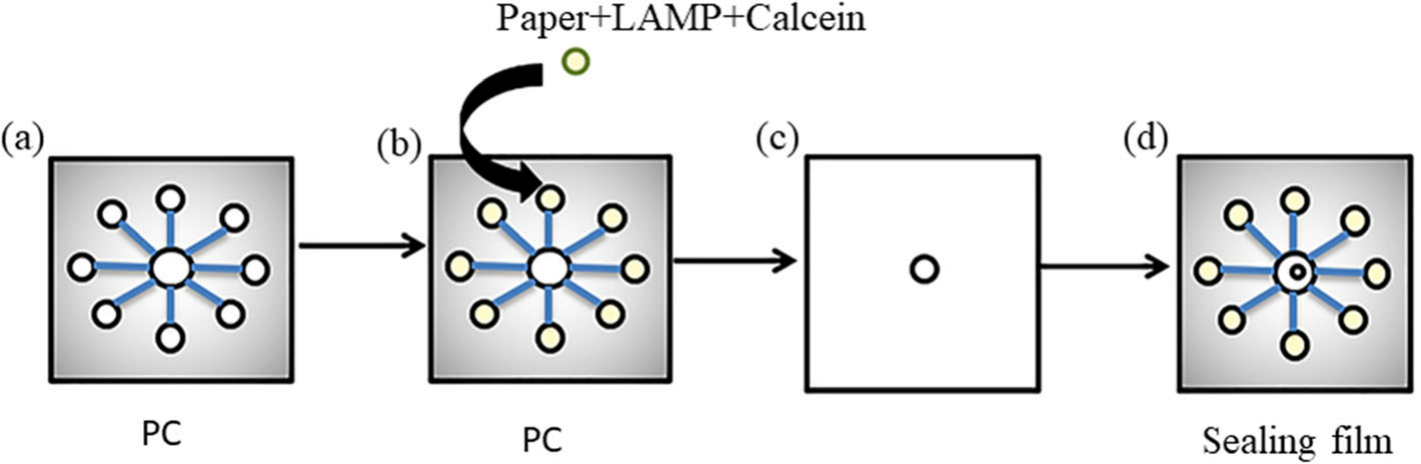
Making process of the microchip. **a** Processed and ready PC sheet; **b** Insert the paper soaked by LAMP reagent and calcein into every reaction chamber; **c** Attach the sealing membrane; **d** Assembled microchip

First, cut the cavity and the channel on the PC plate shown in Fig. 3a. Second, embed the regenerated cellulose paper plate with 0.2 μm pores with LAMP reagent stored in the reaction chamber in Fig. 3b. Third, attach the upper part of the sealing membrane to the top of the PC plate as Fig. 3c. The device is then ready for amplifying various DNA templates from the sample, as Fig. 3d.

**Fig. 4:**
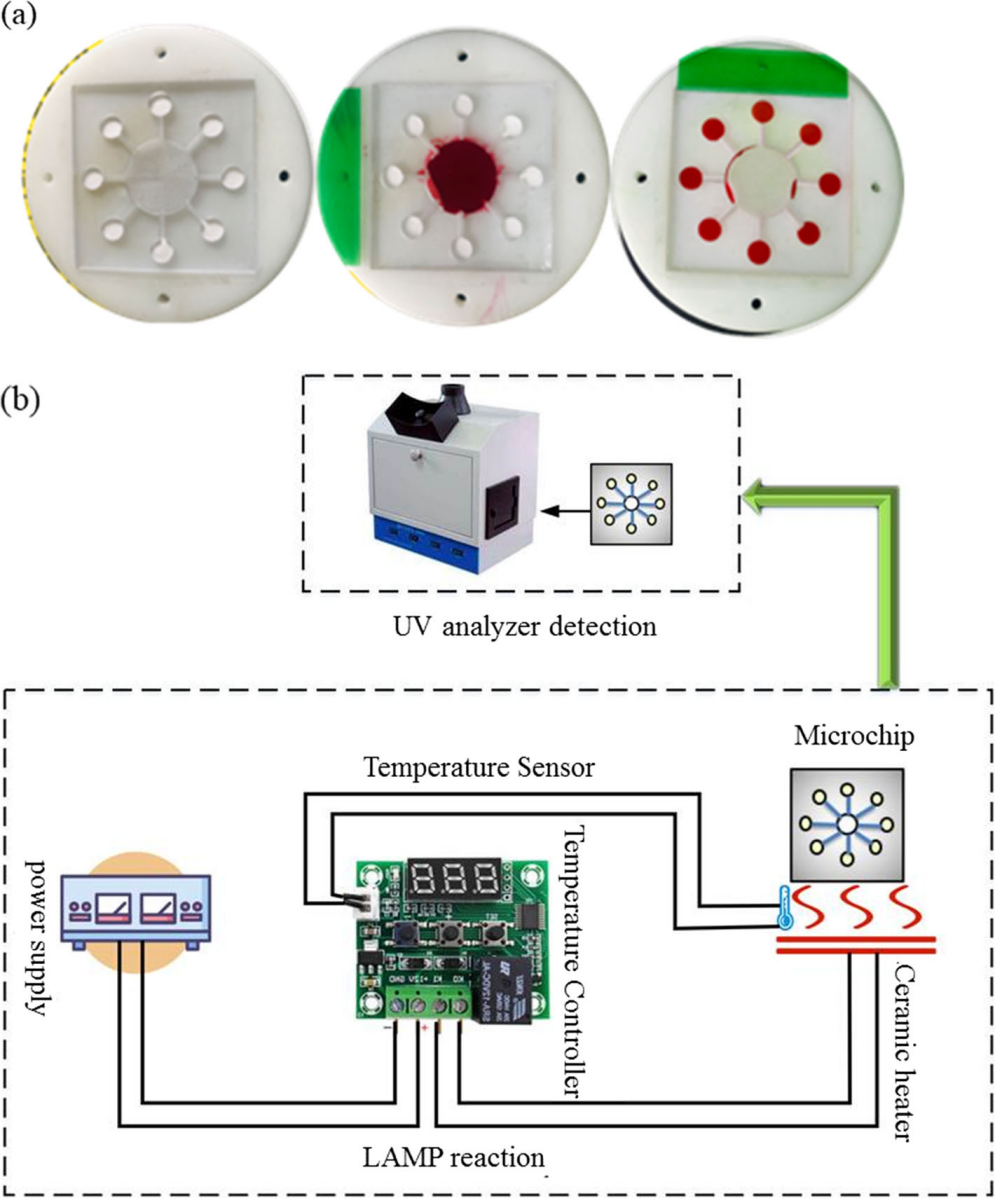
Experiment setup. **a** The process of injecting the red ink solution into the reaction chamber. **b** Schematic diagram of the overall structure of the micro device

Figure 4a shows a photo of the experimental setup showing the rotor and the microchip under actual experimental conditions. The red ink solution is fed into each reaction chamber by applying a rotational speed. The red color eventually appeared on the paper embedded in the chambers.

Figure 4b is a diagram of the overall structure of the micro-device. Power supply, temperature controller, temperature sensor, ceramic heater, microchip, and UV detector. Through testing, we found that the temperature of the ceramic heating plate can be maintained at 65 °C ± 0.1 °C for 30 min, which is enough to complete a LAMP reaction process and meet the temperature requirements of the LAMP reaction on the microchip.

### Multiple channels integrated inspection

**Fig. 5:**
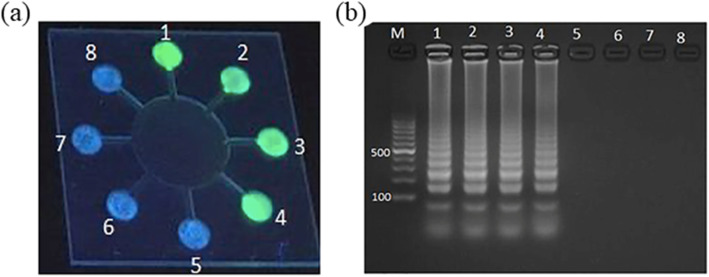
On-chip amplification of the four targets. **a** Microchip images of four foodborne pathogens were detected simultaneously. The #1 ~ #4 chambers contained primer sets for *E. coli O157:H7*, *Salmonella spp., S. aureus*, and *V. parahaemolyticus*, and the #5 ~ #8 chambers were negative controls. **b** Results of gel electrophoresis (the lane number corresponds to the chamber number)

Figure 5a shows the results of a multichannel microchip test in which *E. coli O157:H7, Salmonella spp., S. aureus*, and *V. parahaemolyticus* were sequentially added to the reaction chambers from #1 to #4 in turn, where positive results are indicated by green fluorescence. For comparison, the other four chambers without primers were used as negative controls. The controls have no green fluorescent light emitted. The gel electrophoresis results are shown in Fig. 5b. The samples in chambers #1 ~ #4 developed bright ladder-like bands, while the control chambers had no bands, which further showed consistent results.

### Investigation of sensitivity and specificity

**Fig. 6:**
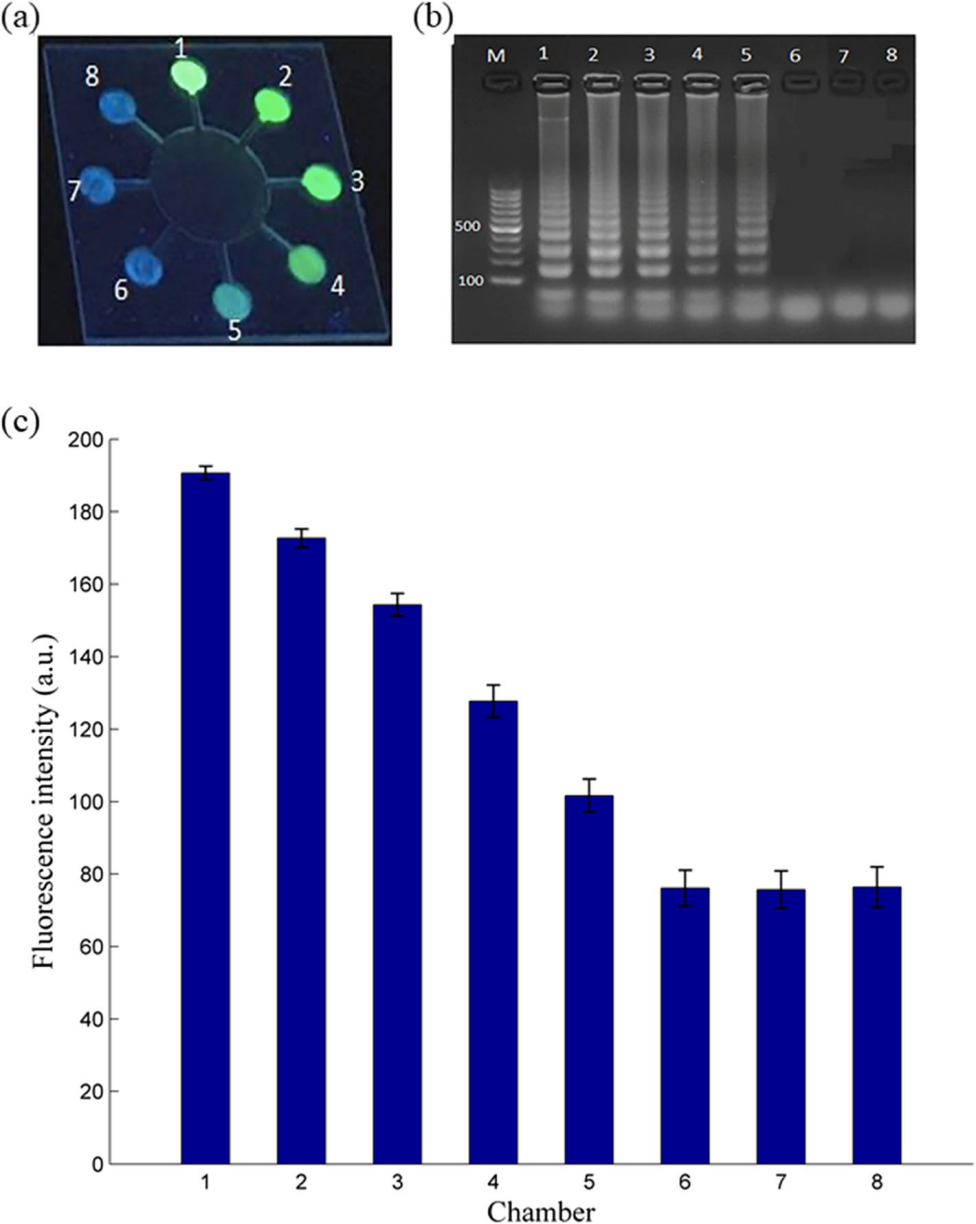
Sensitivity experiment achieved by testing initial serial 10-fold dilutions of the genomic DNA. **a** Image of microchip used for detecting *E. coli O157:H7* DNA in the UV light in the chambers #1 ~ #6, the DN concentration reduces from 134 ng μL^− 1^ to 0.00134 ng μL^− 1^. **b** Chambers #7 and #8 do not contain the DNA template. **c** Results of gel electrophoresis (the lane number corresponds to the chamber number)

Considering the qualitative and quantitative analysis of the microchip ability, we conduct LAMP amplification on *E. coli O157:H7* DNA at different concentrations and then read the result by fluorescent light and gel bands. Figure 6a shows that the micro-device can detect *E. coli O157:H7* DNA concentrations as low as 0.0134 ng μL^−1^. Simultaneously, the result by Agarose Gel Electrophoresis (AGE) is shown in Fig. 6b. In chambers, #1 ~ #6, the *E. coli O157:H7* DNA is in decreasing concentrations from 134 ng μL^−1^ to 0.00134 ng μL^−1^. Moreover, scalariform bands are detected in chambers #1 ~ #5. Figure 6c shows the signal intensity measured using ImageJ software, increasing the *E. coli O157:H7* DNA template concentration. The average intensity from chamber 1 to chamber 6 decreased by about 2.5 folds. In the negative control chambers 7 and 8, the fluorescence signal was negligible.

**Fig. 7:**
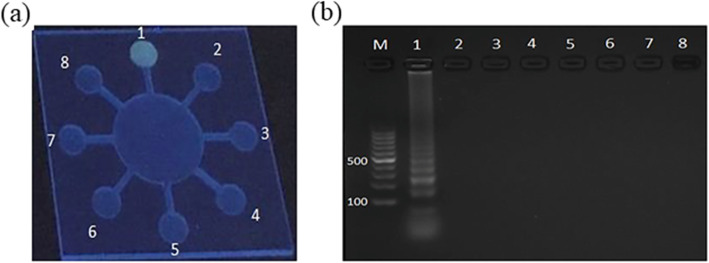
Selectivity of the microchip for *E. coli O157:H7* detection. **a** Image of the microchip in the UV irradiation. Reaction chamber #1 containing *E. coli O157:H7* shows the positive reaction. The chambers #2 ~ #8 containing other bacterial primer groups or containing no primer show the negative reaction. **b** Results of gel electrophoresis (the lane number corresponds to the chamber number)

To study the specificity of the device, a sample of the target template (*E. coli O157:H7*) with a concentration of 0.0134 ng μL^−1^ is injected for the LAMP reaction. As shown in Fig. 7a, no fluorescence signal is detected in the reaction chamber containing primers for *S. aureus, Salmonella spp.*, and *V. parahaemolyticus*. Fluorescence is only detected in the chamber containing *E. coli O157:H7*primers and the template. AGE was used to confirm the specificity of the LAMP test with 3 μL of solution from each reaction chamber. As expected, only the chamber containing *E. coli O157:H7*primers and DNA template show scalariform bands, as shown in Fig. 7b.

### Experimental application of actual milk sample

**Fig. 8:**
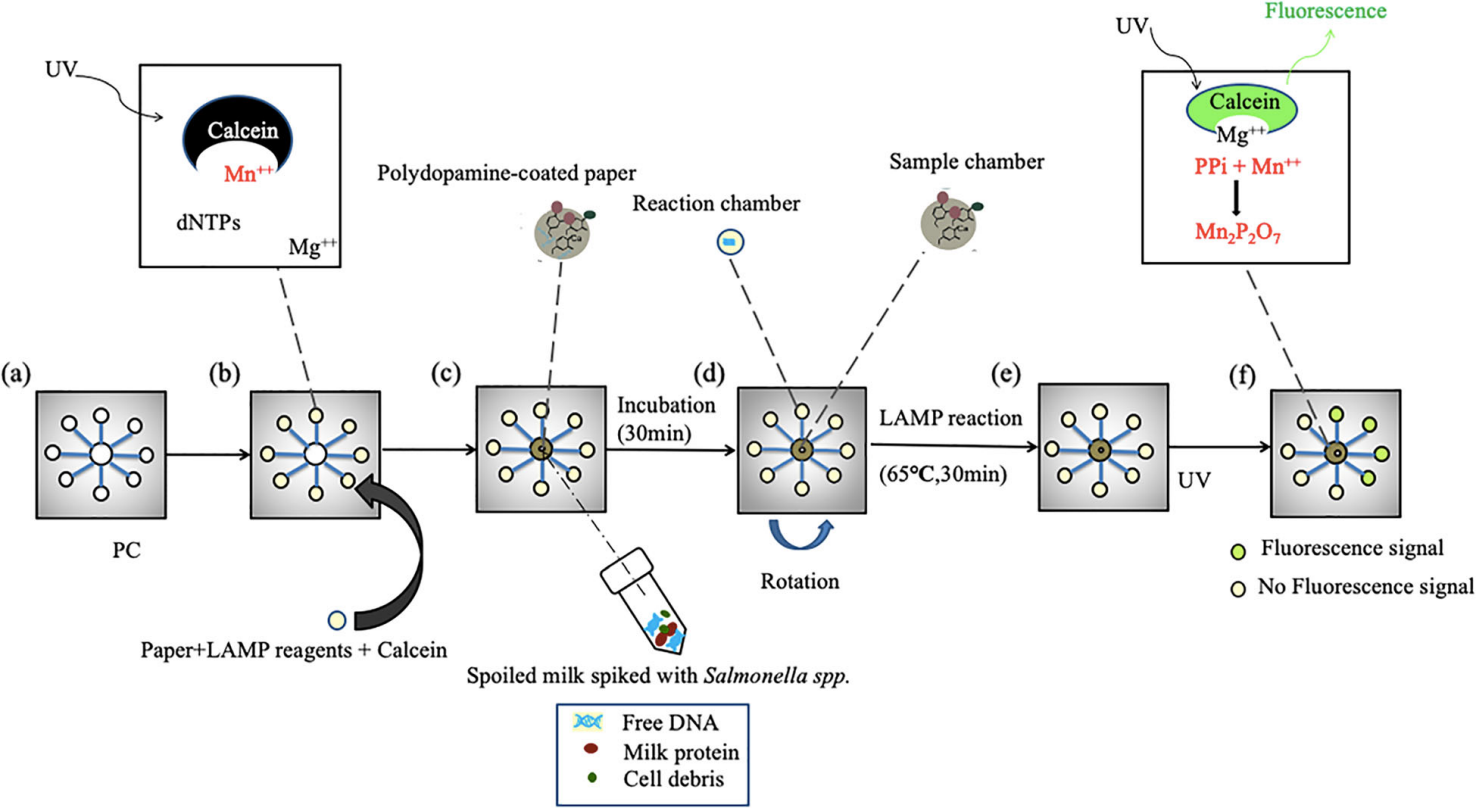
The entire operation workflow. **a** Processed and ready PC sheet; **b** insert the paper soaked by LAMP reagent and calcein in every reaction chamber, where the manganese ion conducts the fluorescence quenching for calcein; **c** extract the DNA of salmonella from the marked milk by using the coated paper on the basis of thermal decomposition and polydopamine; **d** After centrifugation, the polydopamine-coated paper in the sample chamber will capture milk protein and cell debris, and the purified DNA is averagely thrown to the reaction chamber; **e** put the PC microchip on the heater to heat at 65 °C for heating for 30 min; **f** after the LAMP reaction is over, the pyrophosphate ion combines with the manganese ion to release the calcein and thereby the green fluorescence will be emitted in the UV radiation

**Fig. 9:**
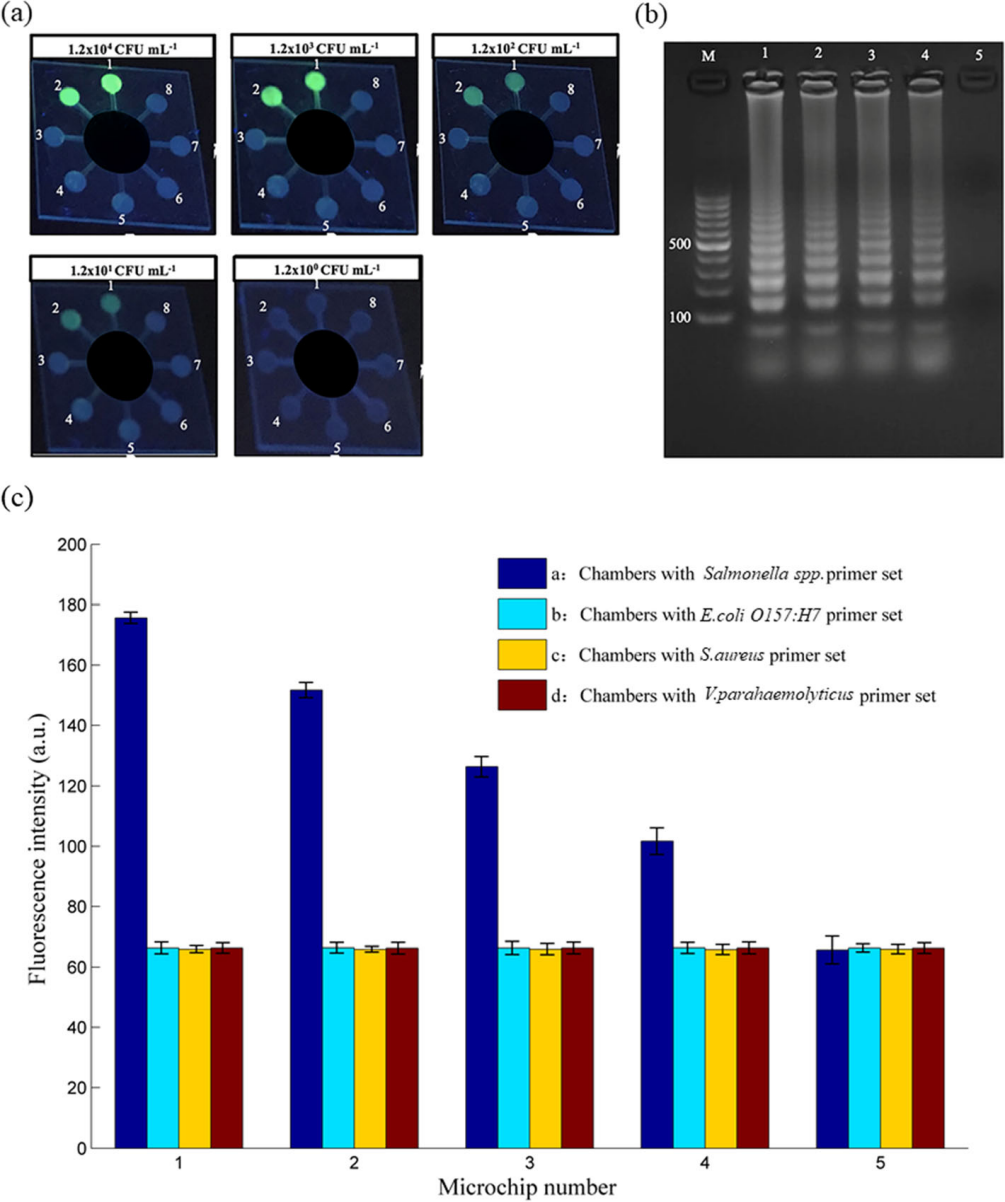
Real sample detection. **a** Fluorescence image of on-chip *Salmonella spp.* detection using spiked milk solutions. Limit of detection (LOD) test by changing the concentration of *Salmonella spp*. . The concentration of bacterium of Salmonella spp. of reaction chambers #1 ~ #5 in turn is:1.2 × 10^4^ CFU mL^− 1^, 1.2 × 10^3^ CFU mL^− 1^, 1.2 × 10^2^ CFU mL^− 1^, 1.2 × 10^1^ CFU mL^− 1^ and 1.2 × 10^0^ CFU mL^− 1^. Chambers #1 and #2 contain the paper plate injected by the *Salmonella spp.* primer groups, #3 and #4 contain the paper plate loaded with the *E. coli O157:H7* primer groups, #5 and #6 contain the paper plate loaded with the *S. aureus* primer groups, #7 and #8 contain the paper plate injected by the *V. parahaemolyticus* primer groups respectively. **b** Results of gel electrophoresis (the lane number corresponds to the PC plate number). **c** A graph shows the fluorescence intensity of each type of bacteria measured under different concentrations of the initial DNA template

Figure 8 shows the whole process of the microchip detecting actual samples. We evaluated the performance of polydopamine-coated paper in DNA extraction and purification. After adding high-temperature heated milk containing Salmonella, incubate at room temperature for 30 min to fully react the bacterial sample with the polydopamine coated paper embedded in the sample chamber. The quinone groups in the polydopamine coated paper reacts with milk components and calcium ions in the solution through Schiff base reactions and chelation reactions, respectively [34]. When the micro-device is rotated, only the purified DNA is transported to each reaction chamber, and the rest of the milk components are trapped in the sample chamber. The paper coated with polydopamine appears to capture potential inhibitors of LAMP. It was confirmed by the appearance of ladder-like bands representing LAMP amplification. Finishing the basic test of the microchip, we successfully detected *Salmonella spp.* in actual food samples. As shown in Fig. 9a, after adding *Salmonella spp.* and diluting to different concentrations, milk samples are figured in below concentrations: 1.2 × 10^4^, 1.2 × 10^3^, 1.2 × 10^2^, 1.2 × 10^1^, and 1.2 × 10^0^ CFU mL^− 1^. The sample with bacteria was injected in the first two chambers for every microchip, and the other six chambers are control groups. The subsequent samples underwent the same LAMP reaction and calcein marking. The results showed that the fluorescence intensity of chambers #1 and #2 is the same for every chip but reduces rapidly as bacterial concentration reduces. The negative controls in chambers #3 ~ #8 show negligible fluorescence signals. The minimum concentration determined from the chips is approximately 12 CFU mL^− 1^. In the practical application of pathogenic bacteria detection when food poisoning occurs, the content of pathogenic bacteria in general samples is above 10^3^ CFU mL^− 1^ as the national standard required. The detection limit of our microchip can meet the requirements of the lowest limit of detection.

Figure 9b and c show that as the number of input bacteria decreases, the fluorescence intensity of the detection signal decreases. The total duration time from sample extraction to fluorescent detect is also illustrated respectively in Table 1. There are approximately 68 min in total, comparable to a previous study [35] with microchip LAMP, which used a centrifugal device rather than a paper-based method.

**Table 1:** Time required for each operation step when performed using the introduced microchip

Procedure	Extraction	Amplification	Detection	Total analysis
Time	35 min	30 min	3 min	68 min

### Discussion

As Fig. 1a shows, the blank lane at 70 °C signifies that LAMP reaction does not occur at 70 °C. According to the bands clearer and brighter at 65 °C than 60 °C, 65°Cis deemed the optimal temperature for the reaction. Similarly, the brightest bands occur at 30 min lane in Fig. 1b, indicating the optimized reaction time.

Thermal denaturation of the DNA template is avoided at the initial stage of LAMP reactions, thus shortening the reaction start time. Because the LAMP amplification template has a dumbbell-shaped structure and contains multiple amplification start points, the amplification process can be conducted simultaneously to improve efficiency. While PCR needs at least 90 min for a complete amplification reaction, LAMP only needs 30 min to complete the reaction, significantly reducing testing time [36].

According to a previous report [37], if the magnesium ion is added to calcein before the LAMP reaction, the green fluorescence of calcein will be quenched, and the dye will become orange. After LAMP amplification, the pyrophosphate and manganese ions generated by the reaction combines and deposit, the magnesium ion will have the opportunity to combine with the calcein and affect the fluorescence signal of calcein. In such a case, the color of a positive detector tube is observed as green fluorescence instead of the initial orange-red color, and a negative detector tube will remain orange-red. Altogether, the final result will be solid green fluorescence in a positive reaction and weak green fluorescence in an adverse reaction when stimulated by 365 nm blue light, as shown in Fig. 2a. As Fig. 2b shows, the results acquired from the paper are consistent with the results from the tube. There is also a fluorescence signal of the paper containing the DNA template.

Furthermore, when acquiring the fluorescence spectrum of the amplified solution as a baseline, there is a significant difference between the tubes before and after the LAMP reaction. A single test can detect four types of foodborne bacterial pathogens via our paper-based LAMP microchips, as Fig. 5 shows, providing a simple and effective substitution test for harmful microbes. These results verify that a microchip is an ideal tool for the rapid mixture of samples and amplification reagents without the need for complicated valves. Additionally, the LAMP test on the microchip provides a more straightforward and more accurate diagnostic tool for detecting multiple pathogens than routine PCR-based methods. Given the experimental properties of LAMP, a single-temperature heater can be used rather than complicated heating equipment.

Additionally, compared with direct detection and tests on LAMP amplicons using SYBR Green I and Fisetin [38], this method dramatically reduces the probability of false positives through the use of calcein. Calcein overcomes the limitations of direct tests on LAMP amplicons. We have known that SYBR Green I and Fisetin require reaction tubes to be opened after the LAMP reaction is complete, introducing the possibility for aerosol contamination. Moreover, SYBR Green I is a latent human mutagen.

The first method is to use fluorescence detection to evaluate the sensitivity of the microchip. After generating the LAMP products and when irradiated by UV light, the pyrophosphoric acid ions in the LAMP solution and Mn^2+^ combine and release calcein, causing the fluorescence to turn from colorless to green. As the concentration of the DNA template is reduced, the pyrophosphate ions are also reduced, leading to the lower fluorescence intensity of positive reactions, which is the principle of the phenomenon in Fig. 6a. AGE is the other method for sensitivity testing, the brightness of the band depends on the DNA concentration. Hence, as the concentration of the DNA template reduces, the brightness of the band reduces accordingly, which is consistent with Fig. 6b. The minimum detection result is the same as the on-microchip detection result. In reaction chambers #6, #7, and #8, there are no prominent scalariform bands, and the visible band is a primer dimer, which is common in the LAMP analysis by AGE [39].

Compared with the traditional PCR method, four primers in the LAMP system must match six or eight specific areas of the target gene to produce a reaction. However, the PCR system needs only one primer in the upstream and downstream regions to match the target gene. For that reason, the LAMP reaction for a sample with *E. coli O157:H7* can only occur in the reaction chamber that contains *E. coli O157:H7* primers, as Fig. 7 presents. It indicates that LAMP has higher specificity [40].

*Salmonella spp.* DNA purified from milk by polydopamine has the following characteristics: the quinone gene group in the polydopamine-coated paper reacts with the milk and calcium ions in the solution via a Schiff base reaction and chelation reaction. When the microchip rotates, the purified DNA is dispersed to each reaction chamber. Simultaneously, the rest of the milk components remain in the sample chamber, as verified by the appearance of scalariform bands indicating LAMP amplicons. That is to say, the paper with polydopamine has the function to remove the inhibitor of LAMP reaction. Furthermore, the results in Fig. 9a, b certifying our microchip can detect the actual sample to ensure there are no foodborne pathogens.

## Conclusions

In summary, our microchip can be used to detect foodborne pathogens with high sensitivity and selectivity. This method has several advantages. First, utilizing the hydrophilic virtue of regenerated cellulose paper, all components except DNA can be loaded onto the paper sheet without the need for complicated pumps or valves of the microfluidic system. Second, the microchip can be reused simply by replacing the paper parts and sterilizing with UV irradiation. Third, the microchip can simultaneously detect multiple samples with high sensitivity and selectivity. For bacterial samples of *E. coli O157:H7*, the sensitivity is 0.0134 ng μL^− 1^. For the detection of *Salmonella spp.* in milk samples, the sensitivity is 12 CFU mL^− 1^. Fourth, it provides direct detection of successful target amplification, enabling faster identification of pathogens (approximately 68 min) than was previously possible. Therefore, the newly developed microchip provides a promising platform to detect multiple targets simultaneously. With appropriate modifications to the reagents, the microchip can be used for nucleic acid analysis in other fields, such as single nucleotide polymorphism identification, genetic diagnosis of clinical samples, and infectious disease monitoring. Besides, due to the convenient design and manufacturing of PC microchips by computer engraving technology, more microchannels and reaction units can be integrated into one chip to detect a large number of DNA targets simultaneously. The LAMP reaction can generate a visible signal based on an increase in calcein fluorescence before the amplification process. In the future, by integrating microfluidic modules (especially DNA extraction modules and optical imaging modules) on a small instrument, the reported method will be generally helpful in the fields of foodborne pathogen detection.

## Method

### Materials

A laser engraver (KB-4060) was bought from Liaocheng Keba Laser Equipment Co., Ltd. (Shandong, China). The fluorescence spectrum was acquired using a NanoDrop 3300 fluorescence spectrometer (Thermo Fisher Scientific, Waltham, MA, USA). Thin polycarbonate (PC) sheets (thickness: 1 mm) were purchased from Shanghai Chenchuang Plastic & Rubber Technology Co., Ltd. (Shanghai, China), sealing membranes were purchased from Rongxin Packaging Material Co., Ltd. (Shenzhen, China). Regenerated cellulose membrane filters with 0.2 μm bore diameter were purchased from the CHMLAB group (Barcelona, Spain). The heater (XH-RP5050) was purchased from Jiangsu Xinghe Electronics Co., Ltd. (Jiangsu, China), and the rotator from Jiangsu Xinkang Medical Equipment Co., Ltd. (Jiangsu, China). The UV Analyzer (ZF-7A) was purchased from Shanghai Qinke Analytical Instrument Co., Ltd. (Shanghai, China). Polydopamine was purchased from Sigma Aldrich (St. Louis, USA). Aseptic paraffin oil was purchased from Hengkang Medical (Hebei, China). LAMP kits containing detection reagent (Bst DNA polymerase and primer), reconstitution fluid (10x isothermal amplification buffer solution, dNTP mixture, 100 Mm MgSO_4_), colorimetric indicator (calcein, including Mn^2+^), a positive control (target bacteria DNA), and a negative control (non-objected bacteria DNA) were purchased from Guangdong Huankai Microbial Sci. & Tech. Co., Ltd. (Guangdong, China). Biowest agarose and loading buffer were purchased from Beijing Solarbio Science & Technology Co., Ltd. (Beijing, China). A fully automatic gel-imaging analysis system (Shanghai, China) was used to test the target band. 100 bp DNA marker and genomic DNA extraction kit were purchased from Takara (Shiga, Japan).

### Bacteria preparation and DNA extraction

*E. coli O157:H7, Salmonella spp., S. aureus*, and *V. parahaemolyticus* were obtained from the Institute of Hygiene and Environmental Medicine (Tianjin, China). *E. coli O157:H7, Salmonella spp., S. aureus,* and *V. parahaemolyticus* were cultured overnight in 5 mL Lysogeny Broth (LB) (37 °C, 200 rpm oscillation). The gDNA was extracted from 1 mL of culture solution using the DNA purification kit. gDNA concentration and mass were determined by UV-visible spectrophotometer and NanoDropTM spectrophotometer. A total of three replicates were performed for each sample. The gDNA was then stored at − 20 °C for future use.

### Optimizing LAMP reaction conditions

To optimize the reaction temperature and LAMP time on the chip, the reaction temperature was adjusted to 60 °C, 65 °C, and 70 °C. The effect of temperature on the response was then determined by the fluorescence intensity obtained. This approach was used to determine the optimal reaction temperature. Optimal reaction time was determined similarly. The reaction time was set to 15 min, 20 min, 25 min, 30 min, and 40 min, and optimal reaction time was also determined based on fluorescence intensity. A total of three replicates were performed for each condition.

### Testing calcein fluorescence

As previously reported [41], mix 25 umol L^− 1^ calcein with 300 umol L^− 1^ manganese chloride to quench the fluorescence of calcein. Add the quenched calcein to the LAMP reagent, shake the solution and observe it under UV radiation. Next, remove the solution and place it in a 65 °C water bath for 30 min, then observe the solution under UV radiation again. To test the fluorescence of calcein on paper, soak the paper in the quenched calcein solution and let it dry at room temperature. After drying, check the paper under UV radiation. Last, use the paper dipped in calcein to test the LAMP byproduct. A total of three replicates were performed for each condition.

### Microchip fabrication

The portable microfluidics chip used for multichannel LAMP testing is made in two layers. The 40 × 40 × 1 mm PC board comprises eight reaction chambers, each having a radius of 2.5 mm. Reaction chambers are connected to the center chamber, which has a radius of 8 mm, by a 5 mm micro-channel that is 0.5 mm deep. The total volume of each reaction chamber and sample chamber is 10 μL and 100 μL, respectively. A computer-aided direct current engraving machine makes the hole at the cavity position, and the sealing membrane seals the upper part of the PC plate.

### Micro-device manufacturing

Use the LAMP kit from Guangdong Huankai Microbial Sci. & Tech. Co., Ltd., which contains primers for the target bacteria. Because there is no need for self-designed primers, the experimental workflow can be further simplified. To amplify and test multiple DNA templates with the device, soak each kind of paper in the reaction chamber with different primers, keep each reaction chamber containing the dry LAMP reagent. Then amplify the paper plate containing different target DNA primers and calcein. Inject the mixed solution containing template DNA of *E. coli O157:H7, S. aureus, Salmonella spp.*, and *V. parahaemolyticus* into the sample chamber via the inlet on the upper layer of the sealing membrane. Then position the device and set the rotator’s velocity to 4000 rpm to uniformly push the sample solution into the reaction chamber via centrifugal force. After completing the rotation step, bring the sample solution to 10 μL in each reaction chamber, then place the device on the portable heater and perform the LAMP reaction at the optimal reaction temperature and time. Store the reagent with the paper plate before placing it in the cavity. Doing so eliminates the need for steps involving sample and reagent injection, which is different from the complicated design of other technologies that rely on different rotating speeds.

### On-microchip LAMP test

Before starting the reaction, place the paper plate containing quenched calcein into the reaction chamber. After the reaction is complete, the pyrophosphate ions and manganese ions combine to show the fluorescence signal of calcein under UV radiation. LAMP amplicons were subjected to AGE for 30 min and then photographed under transparent UV radiation using the Bio-Rad Molecular Imager Gel Chemi Doc XR imaging system.

### Sensitivity and specificity testing

Test sensitivity by conducting a continuous 10-fold dilution of the initial concentration of pathogenic gDNA DNA to determine the sensitivity of the visual inspection of the microfluidic apparatus of the LAMP amplicon. Use the UV-visible spectrophotometer to measure gDNA concentration by the following equation: DNA concentration = F × A260 × molar absorption coefficient (ng μL^− 1^), where F is the dilution ratio of the original DNA solution before measurement and A260 is the absorbency reading at 260 nm. The molar absorption coefficient of double-stranded DNA is 50 ng μL^− 1^. Use only *E. coli O157:H7* gDNA to evaluate the device’s sensitivity and verify the results by AGE for 30 min.

Use the microfluidic device to test the specificity of the LAMP test for gDNA at the lowest detectable concentration based on the sensitivity experiment. Use only the *E. coli O157:H7* gDNA to evaluate the specificity of the device. Place the primers for *E. coli O157:H7*, *Salmonella spp*., *S. aureus,* and *V. parahaemolyticus* in chambers #1 ~ #4, respectively. Inject the template DNA of *E. coli O157:H7* into the central sample chamber and use chambers #5 ~ #8 as negative control chambers. Transfer the solution in the central sample chamber to the reaction chambers via centrifugal force, then heat the device for reaction on the heater at 65 °C for 30 min. Take 3 uL of the reaction solution to verify amplification by AGE after the reaction is complete. A total of three replicates were performed for each condition.

### Using actual samples for on-chip analysis

Insert the paper coated with polydopamine into the central sample chamber to purify DNA from the degenerative milk solution. First, add the *Salmonella spp*. bacteria solution into the milk and incubate at 37 °C for 12 h. Then heat the degenerative milk at 90 °C for 5 min to destroy bacterial cell walls. Next, incubate the solution at room temperature for 30 min to prepare the bacterial sample and polydopamine-coated paper for sufficient reaction. Lastly, apply centrifugal force to distribute the purified DNA solution to each reaction chamber. After loading the sample, conduct the on-chip LAMP reaction and the follow-up fluorescence detection. A total of three replicates were performed for the condition.

## Data Availability

All the data required is included in the manuscript.

## Abbreviations

LAMP: Loop-mediated isothermal amplification
*E. coli O157:H7*: *Escherichia coli O157:H7*
*S. aureus*: *Staphylococcus aureus*
*V. parahaemolyticus*: *Vibrio parahaemolyticus*
WHO: World Health Organization
PCR: polymerase chain reaction
RCA: rolling circle amplification
HAD: helicase-dependent amplification
NASBA: nucleic acid sequence-based amplification
RPA: recombinant enzyme polymerase amplification
EtBr: ethidium bromide
MEMS: micro-electromechanical systems
PC: polycarbonate
UV: ultraviolet
DNA: Deoxyribonucleic acid
AGE: Agarose Gel Electrophoresis
CFU: Colony-Forming units
LOD: limit of detection
DA: Dopamine
PDA: polydopamine
gDNA: Genomic DNA
